# The effects of dual-task cognitive interference on gait and turning in Huntington’s disease

**DOI:** 10.1371/journal.pone.0226827

**Published:** 2020-01-07

**Authors:** Nicollette L. Purcell, Jennifer G. Goldman, Bichun Ouyang, Yuanqing Liu, Bryan Bernard, Joan A. O’Keefe

**Affiliations:** 1 Department of Cell and Molecular Medicine, Rush University Medical Center, Chicago, IL, United States of America; 2 Shirley Ryan Ability Lab, Chicago, IL, United States of America; 3 Northwestern University-Feinberg School of Medicine, Chicago, IL, United States of America; 4 Department of Neurological Sciences, Section of Parkinson Disease and Movement Disorders, Rush University Medical Center, Chicago, IL, United States of America; University of Pennsylvania Perelman School of Medicine, UNITED STATES

## Abstract

Huntington’s disease (HD) is characterized by motor, cognitive, and psychiatric dysfunction. HD progression causes loss of automaticity, such that previously automatic tasks require greater attentional resources. Dual-task (DT) paradigms and fast-paced gait may stress the locomotor system, revealing deficits not seen under single-task (ST). However, the impact of gait “stress tests” on HD individuals needs further investigation. Therefore, the aims of this study were to investigate whether: 1) fast-paced and dual-task walking uncover deficits in gait and turning not seen under single-task, 2) cognitive and gait outcomes relate to fall incidence, and 3) gait deficits measured with wearable inertial sensors correlate with motor symptom severity in HD as measured by the Unified Huntington’s disease Rating Scale-total motor score (UHDRS-TMS). Seventeen HD (55 ± 9.7 years) and 17 age-matched controls (56.5 ± 9.3 years) underwent quantitative gait testing via a 25m, two-minute walk test with APDM^TM^ inertial sensors. Gait was assessed under a 1) ST, self-selected pace, 2) fast-as-possible (FAP) pace, and 3) verbal fluency DT. The UHDRS-TMS and a cognitive test battery were administered, and a retrospective fall history was obtained. During ST, DT, and FAP conditions, HD participants demonstrated slower gait, shorter stride length, and greater lateral step and stride length variability compared to controls (p<0.00001 to 0.034). Significant dual-task costs (DTC) were observed for turns; HD participants took more time (p = 0.013) and steps (p = 0.028) to complete a turn under DT compared to controls. Higher UHDRS-TMS correlated with greater stride length variability, less double-support, and more swing-phase time under all conditions. Decreased processing speed was associated with increased gait variability under ST and FAP conditions. Unexpectedly, participant’s self-reported falls did not correlate with any gait or turn parameters. HD participants demonstrated significantly greater DTC for turning, which is less automatic than straight walking, requiring coordination of body segments, anticipatory control, and cortical regulation. Turn complexity likely makes it more susceptible to cognitive interference in HD.

## Introduction

Huntington’s disease (HD) is an autosomal dominant, neurodegenerative disease caused by an expanded CAG repeat (≥40 repeats, full-penetrance) in the gene for the huntingtin protein (HTT), though symptoms may also occur in some with CAG repeat lengths ≥ 36 (reduced penetrance range) [1]. The expansion results in aggregation of insoluble mutant HTT (mHTT) in neurons, the most vulnerable being striatal medium spiny neurons (MSNs) of the basal ganglia [2]. Ultimately, the proteinaceous aggregates disrupt neuronal function, causing cell death and subsequent motor dysfunction [3]. The progression of HD results in gait and balance impairment which can lead to an increased fall risk [4]. In addition to motor symptoms, cognitive deficits observed in HD make it difficult to focus on a task or divide one’s attention between tasks. As a result, individuals with HD have difficulty responding to multiple stimuli simultaneously [5,6], which can exacerbate motor deficits.

HD progressively impairs automaticity, such that previously automatic tasks, such as walking, begin to require greater attentional resources [7,8]. Cognitive deficits are also prevalent, notably in the domains of executive function, visuospatial processing, processing speed, and short-term memory, which can further exacerbate motor impairments [9,10]. Cognitive-motor dual-task paradigms are a means to assess an individual’s ability to divide their attention during concurrent tasks and have been shown to reveal motor deficits not seen under single-task in Parkinson’s disease (PD) [11], HD [8,12], and multiple sclerosis (MS) [13]. Under DT, individuals with HD have exhibited a decrease in gait speed, cadence, and stride length [8,12] compared to healthy controls, characteristics which contribute to an increased fall risk in other movement disorders [11]. Previously, we found that individuals with HD exhibited significant DT cognitive motor interference for postural stability when vision was removed, and base of support was narrowed [14]. Furthermore, difficulty with dual-tasking is observed in HD during other dual-tasks as well, such that significant dual-task costs (DTC) were observed for speed to perform a cognitive-auditory dual-task [15] and greater finger tap variability during a bimanual motor-motor DT [7].

In addition to DT paradigms, stressing the locomotor systems by asking individuals to modify their pace has been shown to reveal gait deficits in the elderly and other neurodegenerative disorders [16, 17]. A disorder of locomotor timing has been reported in HD with impaired cadence regulation when required to increase their speed, as well as difficulty timing footsteps to an auditory cue [18]. However, the impact of fast-paced gait in HD has been minimally investigated and warrants further study. Therefore, the aims of this study were to investigate whether: 1) fast-paced and dual-task walking uncover deficits in gait and turning not seen under single-task, 2) cognitive and gait outcomes relate to fall incidence, and 3) gait deficits measured with wearable inertial sensors correlate with motor symptom severity in HD as measured by UHDRS-TMS. We hypothesize in HD that 1) fast-paced and dual-task gait will uncover deficits in gait and turning not observed under “normal” walking conditions, 2) greater cognitive impairment in the domains of executive function, visuospatial perception, and processing speed will be associated with greater gait and turning deficits and increased falls, and 3) greater motor symptom severity will be associated with greater gait deficits.

## Materials and methods

### Study participants

This study was approved by the Rush University Medical Center Institutional Regulatory Board (16050204-IRB02). HD participants were recruited from the Rush University Medical Center (RUMC) Movement Disorders HD clinic; age and sex-matched healthy controls were recruited from the community. All participants were co-recruited for a previously published study assessing the effects of dual-tasking and cognition on balance in HD [14]. Inclusion criteria were 1) a clinical diagnosis of HD by a movement disorders/HD expert (JGG) [19], 2) >21 years of age, 3) ability to ambulate for two minutes without an assistive device, and 4) the ability to follow protocol-specific directions with confirmation from family member and/or caregiver. Exclusion criteria included a diagnosis of Juvenile HD, as well as those who have had lower limb or back orthopedic surgery in the past 12 months, or any other disorders negatively affecting gait. Controls were recruited based on the same criteria, with the additional exclusion of cognitive impairment. Participants were classified as having a choreatic, hypokinetic-rigid, or mixed phenotype, as previously described [20]. Informed consent was obtained from all participants in accordance with the RUMC Institutional Regulatory Board.

### Gait assessments

Quantitative gait analysis under self-selected single task (ST), fast-as-possible (FAP) and dual-task (DT) conditions was performed using the well validated, reliable inertial sensor system with gait metrics generated by Mobility Lab^TM^ software (APDM, Oregon, USA) [21]. Six Opal^TM^ wearable sensors were placed on the wrists, dorsum of feet, sternum (2 cm below the sternal notch), and lumbar trunk (at the L5, the approximate center of mass). Participants performed three, 2-minute walk tests [22, 23] on a 25-meter walkway under a 1) self-selected (SS) pace, 2) fast-as-possible (FAP) pace, and 3) cognitive-motor DT condition (DT) at their normal pace, with rests between trials as needed. Participants were instructed to walk at their “normal”, comfortable walking speed for the self-selected and DT trials, and to walk as fast as they safely could, without running, for the FAP trial. During the DT trial, participants were asked to perform an animal naming verbal fluency task, with the instruction that no animal could be repeated. Participants were carefully monitored during all trials for safety. A baseline 2-minute animal naming task was also performed while seated, and this was randomized to be conducted either before or after the gait tests for each participant. This randomization was done to assess whether participants prioritized cognition or gait while dual-tasking. The participants were not told to prioritize the cognitive or motor task during the DT gait condition.

The main outcome variable selected for analysis were 1) cadence (steps/min), 2) stride length (m), 3) gait speed (m/s), 4) swing (% gait cycle), 5) double support (% gait cycle), 6) turn duration (s), 7) number of steps to complete a turn, 8) lateral step variability (m), which groups 3 consecutive steps and derives the extent of perpendicular deviation of the middle foot placement from the first and third step, 9) stride length coefficient of variation (CoV), another common measure of gait variability, and 10) step duration. The extent of DT interference, or the dual-task cost (DTC) in gait and turn performance was defined as DTC (%) = ((DT-ST)/ST)*100, as previously described [24].

### Neuropsychological, balance and clinical rating scale assessments

As previously published [14], the following cognitive battery was administered to evaluate multiple cognitive domains known to be impaired in HD: 1) Montreal Cognitive Assessment (MoCA) (global cognition) [25], 2) Digit Span forwards, backwards, and sequencing (WAIS-IV) (attention and working memory) [26], 3) Symbol Digit Modalities Test (SDMT) (attention and information processing speed) [27], 4) Consortium to Establish a Registry for Alzheimer’s disease (CERAD Word List Memory, delayed recall portion) (memory recall) [28], 5) Judgment of Line Orientation (JLO) (visuospatial perception) [29], and 6) Animal naming (verbal fluency) [30]. The Unified Huntington’s disease Rating Scale total motor score (UHDRS-TMS) was administered by a movement disorder/HD neurologist (JGG) [19]. Higher UHDRS-TMS values reflect the presence of more severe motor symptoms. The Berg Balance Scale (BBS) [31] and the Activities-Specific Balance Confidence Scale (ABC) [32] were used to assess participants’ awareness of their postural stability and collect performance-based balance information. Higher scores on all cognitive tests and functional balance scales are indicative of better performance. Additionally, participants were asked to recall how many falls they had in the past 12 months.

### Statistical analyses

Clinical characteristics were compared between the HD and healthy control group using two-tailed Student t-tests for parametric and normally distributed measures, or the Mann-Whitney U test for variables that did not have normal distributions. Differences in gait variables and animal naming cognitive assessment under ST and DT conditions, and the DTC for each of the primary outcome variables between HD participants and healthy controls were examined with the same statistical tests. For those gait variables observed to be significantly impaired in HD under all conditions compared to controls, a repeated-measures analysis of variance (ANOVA) (parametric) or a Friedman test (non-parametric) with Bonferroni post-hoc tests were performed to assess if those impairments were further exacerbated under FAP and DT conditions.

Correlations between gait and turn measures and cognitive test scores, UHDRS-TMS, and retrospective falls were examined in the HD group using Spearman’s rho. The statistical significance for these comparisons was set at p = 0.05 given the exploratory nature of this work. However, due to the large number of variables tested and correlations performed, the correlations that remained significant after Bonferroni corrections using an adjusted p-value of < 0.001 were also indicated with a ‘b’ superscript. An exploratory linear regression analysis was then performed incorporating disease duration and UHDRS-TMS as covariates to further investigate potential cognitive and gait relationships.

## Results

### Participant characteristics

Seventeen individuals with HD and 17 age-matched healthy controls were enrolled in a larger dual-task study examining the effects of cognition on balance and gait in Huntington’s disease; participant demographic and clinical characteristics are listed in Table 1. UHDRS-TMS scores ranged from 7–39. Five of the seventeen HD participants were not on any medications at the time of testing. The most common medications reported were an NMDA antagonist (n = 4), benzodiazepine (n = 4), cholinesterase inhibitor (n = 4), antipsychotic (n = 2), and selective serotonin reuptake inhibitors (SSRI) (n = 2). No participants were taking vesicular monoamine transporter 2 (VMAT2) inhibitors. Eight HD participants were characterized as having a mixed phenotype, 4 as choreatic, and 4 as hypokinetic-rigid, one participant did not have a UHDRS-TMS recorded. Ten out of the 17 HD individuals reported having >1 fall in the past twelve months. HD participants showed significant deficits in global cognition (MoCA, p = 0.0009), response inhibition (Stroop, p = 0.007), attention/processing speed (SDMT, p<0.0001), verbal fluency (animal naming ST and DT, p<0.0001), visuospatial processing (JLO, p = 0.0083) and working memory (Digit Span, p = 0.0087) compared to controls. HD participants’ delayed recall (CERAD Word List) performance was not significantly different from controls. There were no significant DTC for the animal naming cognitive task during the two-minute walk (DT) compared to the ST while seated in HD subjects compared to controls. HD participants reported lower balance confidence on the ABC (p = 0.0001), worse scores on the BBS (p<0.0001), and a greater number of falls within the past twelve months (p = 0.0007) compared to controls (Table 1).

**Table 1:** Subject characteristics.

	Healthy controls (n = 17)	Huntington’s disease (n = 17)
Age (years)	56.47 ± 9.30 (37–69)	55 ± 9.66 (36–67)
Sex	8 Females, 9 Males	7 Females, 10 Males
BMI (kg/m)	26.29 ± 5.22 (20.8–37.8)	24.68 ± 3.79 (17.80–31.00)
Years of education	16.59 ± 2.82	15.59 ± 2.67
UHDRS-Total Motor Score	----	21.86 ± 9.86 (7–39)
Trunk Chorea	----	0.69 ± 0.79 (0–2)
Trunk, upper & lower extremity chorea score	----	0.94 ± 0.66 (0–2)
Disease Duration (years)	----	5 ± 2.8 (3–13)
One-year retrospective Falls (#)	0.176 ± 0.529 (0–2)	**2.29** **±** 2.69 (0–10)***
MoCA	26.47 ± 2.79 (20–30)	**22.70** **±** 3.46 (12–28)***
SDMT	99.34 ± 13.42 (80.4–131.1)	**70.89** **±** 20.74 (45.5–105.9)****
Stroop -CW	45.5 ± 8.36 (35–59)	**37.19** **±** 7.89 (25–52)**
CERAD-Recall	6.35± 1.69 (4–10)	5.59 ± 2.24 (2–10)
JLO	12.35 ± 1.87 (8–15)	**10.06** **±** 2.79 (5–14)**
Digit Span	11.12 ± 2.47 (5–14)	**8.23** **±** 3.45 (1–15)**
Animal Naming-ST (#)	37.41 ± 8.44 (20–51)	**21.76** **±** 9.73 (8–53)****
Animal Naming-DT (#)	35.06 ± 7.96 (21–49)	**20.71** **±** 10.25 (6–53)****
DTC Animal naming (% change)	-4.75 ± 16.18 (-35.14–31.82)	-5.44 ± 19.16 (-39.13–33.33)
ABC	95.38 ± 5.05 (83.7–100)	**81.20** **±** 13.2 (50.31–100)***
BBS (0–56)	55.88 ± .33 (55–56)	**51.18** **±** 3.15 (44–56)****

All values are mean ± SD with range in brackets unless indicated otherwise. Key: Body Mass Index (BMI), Unified Huntington’s Disease Rating Scale-total motor score (UHDRS-TMS), Activity Specific Balance Confidence scale (ABC), Berg Balance Scale (BBS), 1 year fall history (# self-reported in last year), Montreal Cognitive Assessment (MoCA), Symbol Digit Modalities Test (SDMT), Stroop, Color-Word (CW), Consortium to Establish a Registry for Alzheimer’s disease (CERAD), Judgment of Line Orientation (JLO), and Digit Span values were compared between Huntington’s disease subjects and controls. The SDMT, Stroop-CW, CERAD-Recall and Digit Span were scaled to the subject’s age and years of education. Note that this table was published in a previous balance paper using the same HD cohort [14].

Significant differences are bolded.

*p < 0.05

** p< .01

*** p< 0.001

**** p < 0.0001

### Gait: Single task, fast-as-possible, and DT conditions

**Fig 1:**
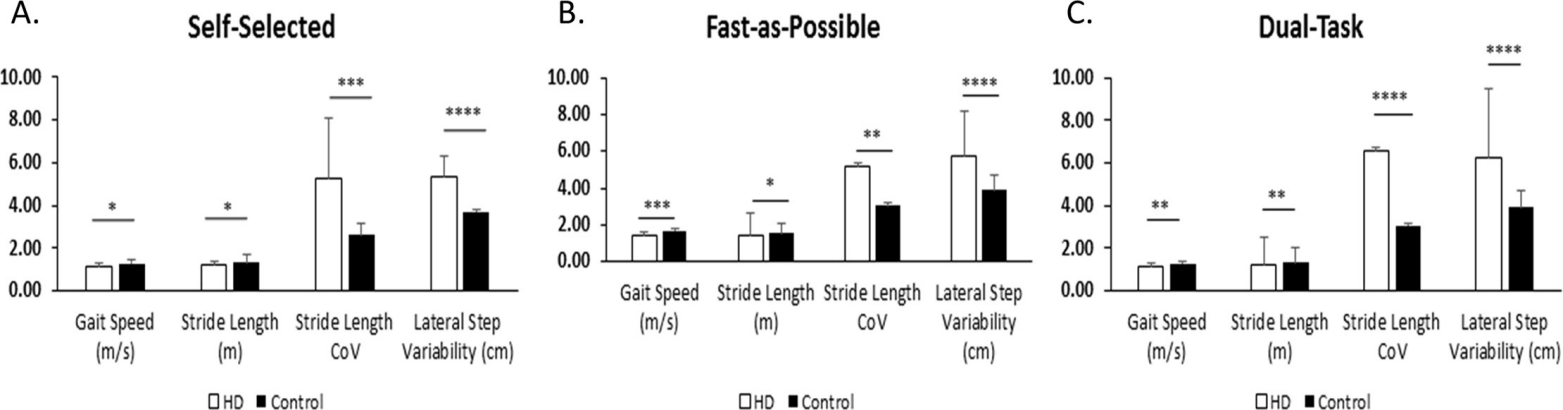
Gait parameters under SS, FAP, and DT conditions. Significantly different gait parameters of HD individuals compared to healthy controls under SS (A), FAP (B), and DT (C) conditions. *p < 0.05 ** p< .01, *** p< 0.001, **** p < 0.0001.

Independent sample t-tests indicated there was not a significant difference between the left and right foot for all gait parameters; therefore, the average was calculated and used for all future analyses. Four spatiotemporal gait parameters were consistently observed to be significantly different between the HD and control groups under all three conditions (SS, FAP, DT); these were gait speed, stride length, lateral step variability, and stride length variability (CoV) (Fig 1). HD participants exhibited significantly slower gait speed (p = 0.034, 0.0005, 0.004), shorter stride length (p = 0.0004, 0.029, 0.005), and greater lateral step variability (p < 0.00001, 0.003, < 0.00001) and stride length variability (p = 0.00001, 0.001, 0.00001) compared to controls (Table 2). Additionally, HD individuals took longer to complete a turn compared to controls under the FAP condition (p = 0.045). Compared to SS pace trials, HD individuals were able to significantly increase their gait speed under FAP conditions (p < 0.00001); no difference was observed during DT. Stride length was observed to increase during FAP conditions compared to SS (p < 0.00001); no difference was seen under DT. Lateral step variability increased during DT conditions compared to SS (p = 0.0026); no difference was seen under the FAP condition. After correcting for multiple comparisons, stride length variability was not significantly different between conditions. No significant differences were found for turning across conditions in HD. Although controls increased their gait speed (p < 0.00001) and stride length (p = 0.00002) during FAP trials compared to SS trials, no significant differences were observed for lateral step and stride length variability between conditions and no differences were observed between DT and SS conditions.

**Table 2:** Gait and turning during SS, FAP, and DT conditions.

	Self-Selected		Fast-as-Possible		Dual-Task	
	Control	HD	Control	HD	Control	HD
**Gait Rhythm**						
Cadence (steps/min)	113.93 ± 7.13	111.63 ± 11.31	131.41 ± 8.52	124.28 ± 14.33	115.0 ± 6.33	111.5 ± 13.33
**Pace**						
Gait Speed (m/s)	**1.28** **±** **0.169**	**1.14** **±** 0.188*	**1.67** **±** **0.16**	**1.44** **±** 0.18***	**1.28** **±** **0.12**	**1.11** ±0.19**
Step Duration (s)	0.53 ± 0.033	0.54 ± 0.057	0.46 ± 0.03	0.49 ± 0.054	0.52 ± 0.029	0.55 ± 0.67
Stride Length (m)	**1.34** **±** **0.133**	**1.23** **±** 0.155*	**1.52** **±** **0.159**	**1.40** **±** 0.157*	**1.33** **±** **0.12**	**1.19** **±** 0.15**
**Gait Cycle Phase**						
Double Support (% gait cycle)	18.97 ± 2.95	19.79 ± 3.47	15.33 ± 3.25	17.01 ± 2.91	19.43 ± 2.82	19.8 ± 3.64
Swing (% gait cycle)	40.52 ± 1.49	40.13 ± 1.76	42.54 ± 1.92	41.62 ± 1.58	40.27 ± 1.41	40.14 ± 1.86
**Gait Variability**						
Lateral Step Variability (cm)	**3.70** **±** **0.53**	**5.36** **±** 0.99****	**3.88** **±** **0.58**	**5.71** **±** 1.24****	**3.91** **±** **0.74**	**6.21** **±** 1.39****
Stride Length CoV	**2.61** **±** **0.35**	**5.24** **±** 2.95****	**3.01** **±** **0.84**	**5.22** **±** 2.55***	**3.08** **±** **0.79**	**6.55** **±** 3.35****
**Movement Transition**						
Turn Duration (s)	2.07 ± 0.32	2.03 ± 0.28	1.77 ± 0.24	**1.95** **±** 0.26*	1.88 ± 0.24	2.07 ± 0.32
Steps to Turn (#)	3.70 ± 0.55	3.58 ± 0.60	3.57 ± 0.57	3.73 ± 0.46	3.37 ± 0.61	3.66 ± 0.73

Gait and turning parameters of the control and HD group under self-selected (SS), fast-as-possible (FAP), and dual-task (DT) conditions. Data reported as mean ± SD. Significant differences are bolded.

*p < 0.05

** p< .01

*** p< 0.001

**** p < 0.0001

**Fig 2:**
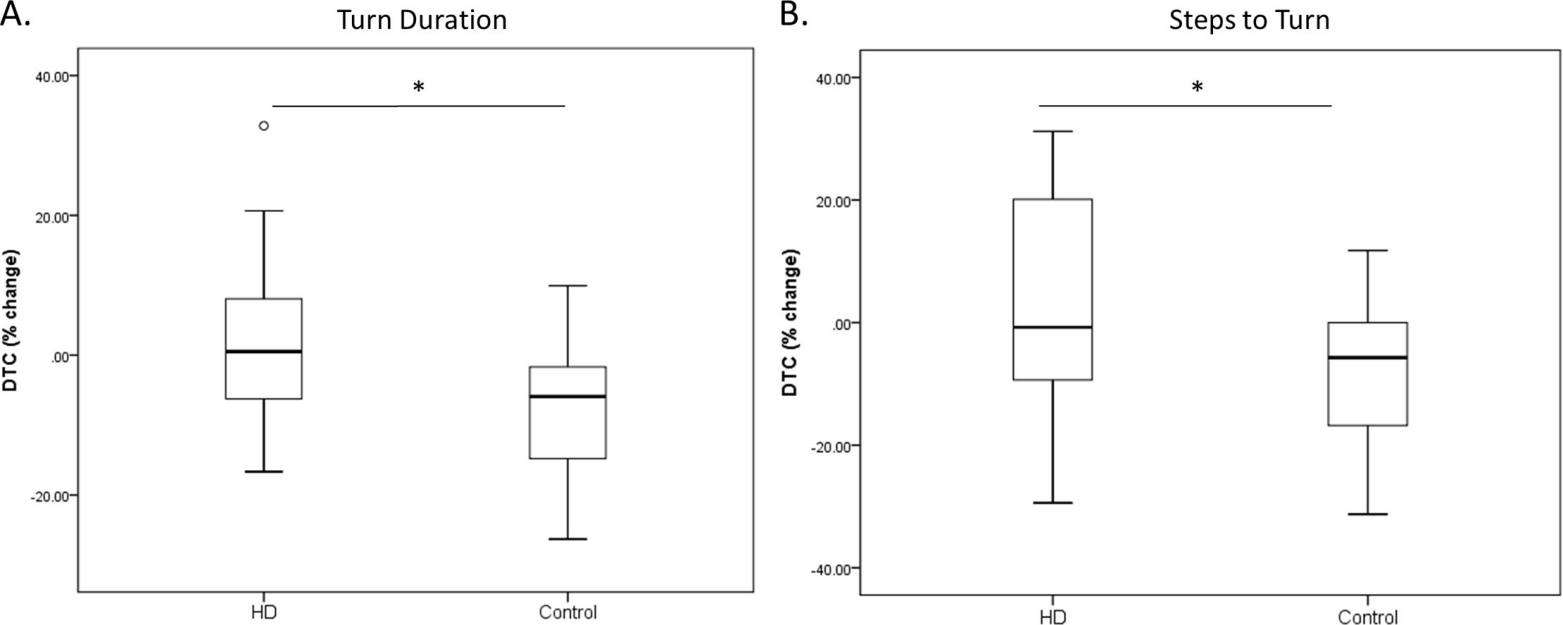
Dual-task costs while turning. Dual-task costs (DTC) of HD participants during turns; Results of a two-tailed independent sample t-test indicating significant cognitive interference observed for turn duration (p = 0.013) (A) and number of steps to turn (p = 0.029) (B) in HD compared to controls. Center line indicates the median value (50^th^ percentile), bottom line of the box represents the 25^th^ percentile, top line of the box represents the 75^th^ percentile, and the whiskers represent the maximum and minimum values, with the exception of one outlier in the HD group for turn duration. DTC calculated as ((DT-ST)/ST)*100. * p < 0.05.

The only significant DTC in HD participants were found for turn variables such that the HD group exhibited significantly greater turn durations (p = 0.013) (Fig 2A) and more steps to complete a turn (p = 0.029) (Fig 2B), than controls.

### Correlations between gait, cognition, UHDRS-TMS, and falls

**Fig 3:**
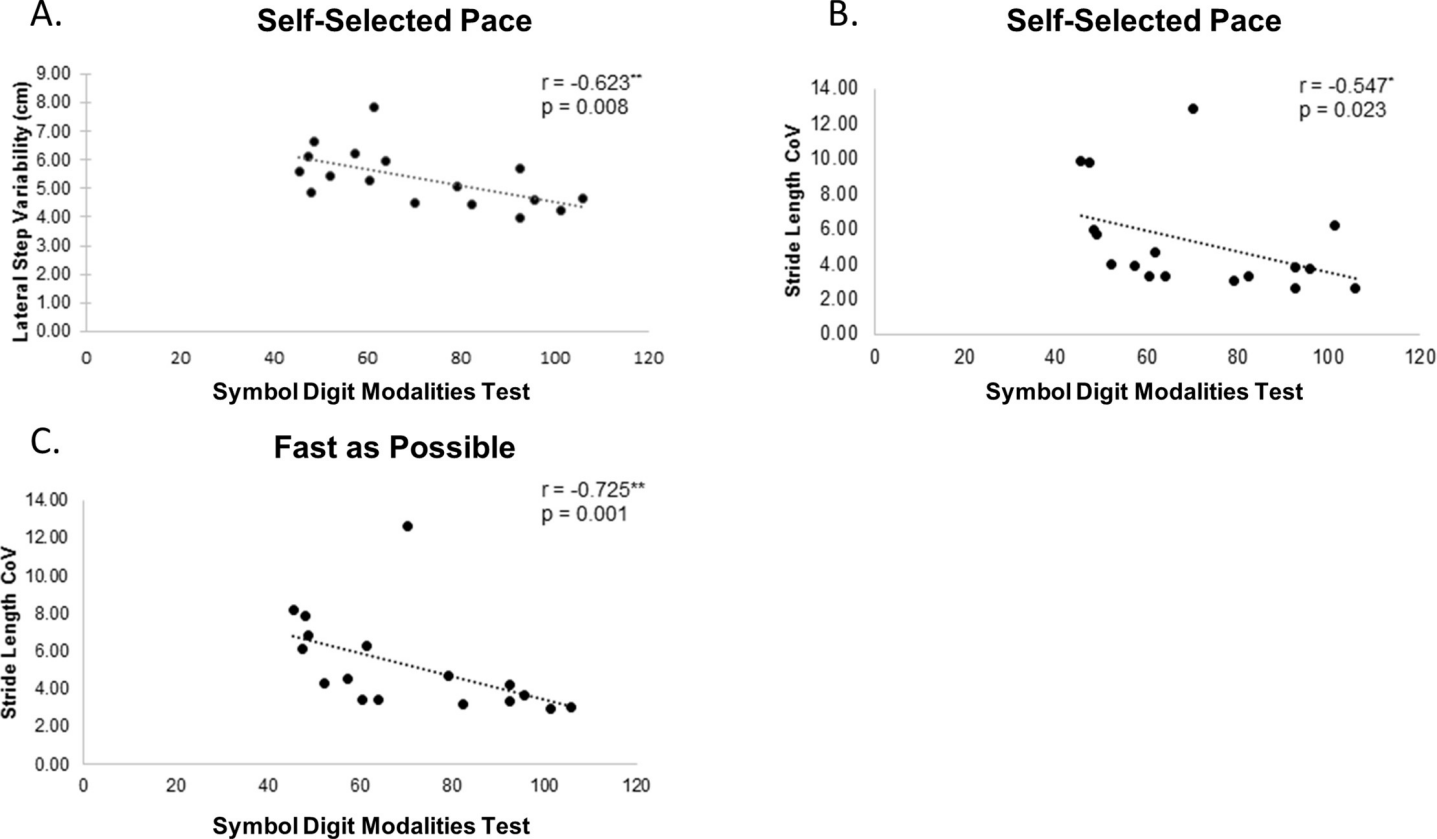
Cognition and gait associations. Cognition and gait correlations in HD. Lower scores on the symbol digit modalities test (SDMT) are associated with greater lateral step variability during SS trials (A) and greater stride length variability under SS (B) and FAP (C) trials. *p < 0.05, ** p< .01.

Correlations with cognitive tests were limited to our cognitive domains of interest: executive function (Digit Span, Stroop and animal naming), information processing speed (SDMT), and visuospatial processing (JLO). Poorer performance on the SDMT and animal naming was significantly associated with increased gait variability in individuals with HD. Lower SDMT scores were associated with increased lateral step variability under SS pace (r = -0.623, p = 0.008) (Fig 3A), as well as increased stride length CoV under SS (r = -0.547, p = 0.023) (Fig 3B) and FAP (r = -0.725, p = 0.001) (Fig 3C) conditions. Furthermore, poorer performance on animal naming was associated with greater stride length CoV during the FAP trials (r = 0.706, p = 0.002). When the significant cognitive variables were entered into the exploratory regression model controlling for disease duration and UHDRS-TMS, the significant associations between cognition and gait were no longer observed.

UHDRS-TMS were positively correlated with percent of time spent in swing phase under SS (r = 0.608, p = 0.013), FAP (r = 0.678, p = 0.004), and DT (r = 0.696, p = 0.003) conditions. Additionally, UHDRS-TMS were negatively correlated with percent of time spent in double support for all three conditions (SS: r = -0.610, p = 0.012; FAP: r = -0.670, p = 0.004; DT: r = -0.672, p = 0.004). Higher UHDRS-TMS, indicating greater clinical severity, was also associated with increased stride length CoV under SS (r = 0.504, p = 0.046), FAP (r = 0.753, p = 0.001 ^b^) and DT (r = 0.6382, p = 0.004) conditions (Table 3). No gait or cognitive variables were found to be correlated with the number of falls self-reported in the past 12 months. All p-values with a ‘b’ superscript indicate the associations are still significant after applying Bonferroni corrections with an adjusted p-value of 0.001.

**Table 3:** Relationship between UHDRS-TMS and gait variables.

Self-Selected	UHDRS-TMS	Fast as Possible	UHDRS-TMS	Dual-Task	UHDRS-TMS
**Gait Rhythm**		**Gait Rhythm**		**Gait Rhythm**	
Cadence	r = -0.215 p = 0.425	Cadence	r = 0.084 p = .757	Cadence	r = -0.324 p = 0.220
**Pace**		**Pace**		**Pace**	
Gait Speed	r = -0.240 p = 0.931	Gait Speed	r = 0.193 p = 0.474	Gait Speed	r = -0.152 p = 0.574
Step Duration	r = 0.203 p = 0.451	Step Duration	r = -0.053 p = 0.845	Step Duration	r = 0.353 p = 0.180
Stride Length	r = 0.112 p = 0.679	Stride Length	r = 0.151 p = 0.578	Stride Length	r = -0.158 p = 0.560
**Gait Cycle Phase (% time)**		**Gait Phase Cycle (% time)**		**Gait Phase Cycle (% time)**	
Double support	r = -0.610* p = 0.012	Double Support	r = -0.670** p = 0.004	Double Support	r = -0.672** p = 0.004
Swing	r = 0.608* p = 0.013	Swing	r = 0.678** p = 0.004	Swing	r = 0.696** p = 0.003
**Gait Variability**		**Gait Variability**		**Gait Variability**	
Lateral Step Variability	r = 0.394 p = 0.132	Lateral Step Variability	r = 0.05 p = 0.854	Lateral Step Variability	r = 0.246 p = 0.358
Stride Length CoV	r = 0.504 * p = 0.046	Stride Length CoV	r = 0.753** p = 0.001 ^b^	Stride Length CoV	r = 0.682** p = 0.004
**Movement Transition**		**Movement Transition**		**Movement Transition**	
Turn Duration	r = -0.215 p = 0.423	Turn Duration	r = -0.098 p = 0.718	Turn Duration	r = 0.083 p = 0.761
Steps to Turn	r = 0.081 p = 0.765	Steps to Turn	r = -0.110 p = 0.685	Steps to Turn	r = 0.112 p = 0.679

Correlations between motor symptom severity, as measured by the Unified Huntington’s disease rating scale–total motor score (UHDRS-TMS), and gait variables. Correlation coefficient (r) and p-values reported under all three gait conditions. The ‘b’ superscript indicates the associations still significant after applying Bonferroni corrections with an adjusted p-value of 0.001.

## Discussion

Our study found that cognitive interference is significant when individuals with HD are turning during the gait cycle. These results highlight the complexity of turning and how modifying gait patterns to complete a turn requires both motor and cognitive resources [33]. Turning is believed to rely on frontal lobe functioning significantly more than straight walking, so that the negative effects of cognitive impairment on the postural adjustments of turning are more pronounced [33]. Additionally, turning necessitates more inter-limb and spinal segment coordination and is greatly impacted by cognitive functioning, such as processing speed [34, 35], which is notably impaired in HD [10]. This knowledge is in alignment with our finding of significant cognitive interference during turns in HD. These results expand upon our previous findings where individuals with HD exhibited significant cognitive interference under environmental conditions that markedly challenge postural stability [14]. In addition, a previous study in HD indicated that multitasking was the most reported cause of falls [36], supporting the theory that cognitive deficits, in combination with loss of automaticity, can result in decreased postural stability in HD, especially during the postural adjustments required for turning.

The fact that there were no elevated DTC for spatiotemporal aspects of gait or cognition during straight walking suggests that the HD group as a whole subscribed to the “posture-second” strategy of dual-tasking observed in PD, where both elements of the DT are treated with equal attention [37–39]. Employing this strategy becomes an issue though, due to limited cognitive resources and impaired postural control, resulting in neither task being adequately accomplished [37].

Interestingly, the same four spatiotemporal gait parameters were consistently found to be impaired in HD compared to healthy controls under all testing conditions: gait speed, stride length, lateral step variability, and stride length variability. The gait domains of pace and variability are commonly reported as abnormal in previous gait studies in other movement disorders [8, 40–42]. However, a number of gait variables are included in these domains and a set of sensitive variables for the HD population has not been validated. Lateral step variability is not a commonly reported outcome measure, although our study suggests it should be investigated in the future, as it was a notable abnormal feature of HD participants. Gait variability is reported to be increased in HD [36,43,44] and is thought to be a result of a disruption of the basal ganglia’s cueing mechanism to the supplementary motor cortex [18]. Disrupted cueing can then cause inefficient generation of movement timing and greater movement variability, increasing fall risk [18]. These findings lay the groundwork for future, more targeted gait studies in HD, suggesting that gait speed, stride length, stride length variability, and lateral step variability could all be sensitive outcome measures for future clinical trials.

We found that DT gait in HD, but not healthy controls, increases lateral step variability. Increased gait variability has been shown to be associated with increased gait instability and falls in other populations [45]. Although we did not find gait variability parameters to be associated with falls in the present study, perhaps due the low sample size, further prospective studies with larger subject numbers might find that increased gait variability may potentially serve as a marker or predictor of future fallers and thus have clinical utility.

A reduction in attention and information processing speed, as assessed by the SDMT, were found to be associated with greater gait variability in HD. Prior studies have indicated that lower processing speed is associated with impaired stability and increased falls in MS [46], impaired turning in PD [35], slower gait speed in an aging population [47], and worse gait and balance as assessed by the Tinetti Mobility Test in HD [9]. However, our observed association between cognition and gait was no longer significant after controlling for disease duration and UHDRS-TMS in our exploratory regression analysis. We posit that this was due to our low sample size. Future studies with larger subject numbers, as well as obtaining a total UHDRS score in order to have a more accurate measure of disease severity, will hopefully allow us to determine the impact of cognition and disease severity on gait deficits in HD.

Similar to the findings of our balance study in HD [14], we did not find the number of retrospective falls to correlate with any cognitive and gait parameters in HD individuals. We believe the lack of retrospective fall correlations can be attributed to the small sample size of this study. Additionally, retrospective fall reporting relies on a participant’s memory recall and self-awareness and individuals with HD often exhibit a lack of disease insight, making a self-reported fall history prone to under-reporting. Therefore, prospective recording, caregiver corroboration, or a fall detection monitoring device would provide a more accurate fall report. Subsequent studies will include a larger cohort with more accurate means of reporting falls or prospective fall reporting to strengthen this analysis.

Previous studies in HD, PD, and cerebellar ataxia reported that participants spent more time in double support and stance phase and less time in swing phase [48–53] as a possible compensatory measure to maintain postural stability [52,53]. However, we did not find this to be the case in our HD cohort; swing and double support time were not significantly different from controls under any gait condition. We attribute these findings to the variability of motor symptoms in our HD group.

Increased motor symptom severity was associated with greater gait variability suggesting those with greater motor impairment have a more unstable gait. Higher UHDRS-TMS, indicating greater motor symptom severity, were significantly correlated with less time in double support and more time in swing phase. These results are difficult to explain but suggest further investigation into the relationship between gait and motor severity. Future studies utilizing wearable inertial sensors might aid in better characterization of choreatic gait and the poorly defined “stutter step” gait pattern exhibited by some individuals with HD [54]. The relationship we observed between gait and UHDRS-TMS is difficult to interpret due to the variability of gait impairment within our sample. Therefore, more studies with larger subject numbers and stratification based on motor severity are needed to create a thorough spatiotemporal and kinematic profile of HD gait and how gait relates to HD motor severity.

The strengths of this study include: 1) the use of a sensitive inertial sensor system to assess gait in HD under challenging conditions reflective of everyday scenarios, 2) significant contributions to the growing body of work characterizing the impact of cognition and cognitive dual-tasking on turns during ambulation in HD and 3) the use of an extensive neuropsychological test battery to assess multiple cognitive domains and their interaction with gait deficits. This study is not without limitations. As previously mentioned, future studies will have larger subject numbers and include participants with varying severities of motor and cognitive symptoms to potentially stratify HD participants and examine phenotypic differences in dual-task capabilities and costs. Future studies would also benefit from complementary neuroimaging or neurophysiological data to understand the neural mechanisms underlying gait control in HD and how basal ganglia cortical connectivity, volumetric changes, and/or activation patterns relate to turn deficits and dual-tasking.

## Conclusion

In conclusion, individuals with HD exhibit detrimental effects of cognitive interference while turning, highlighting the complexity of turning and the dynamic motor and cognitive coordination necessary to safely complete a turn. Additionally, impaired attention and processing speed was associated with more gait variability and is a domain that should be investigated further as an indicator of fall risk in HD. More thorough studies need to be done to quantitatively characterize the choreatic gait pattern and distinguish it from other movement disorders, improving clinical gait assessment in patients with HD.
